# PIF3 Is Involved in the Primary Root Growth Inhibition of *Arabidopsis* Induced by Nitric Oxide in the Light

**DOI:** 10.1093/mp/sst142

**Published:** 2013-10-21

**Authors:** Sulan Bai, Tao Yao, Miaomiao Li, Xiaomin Guo, Yaochuan Zhang, Shengwei Zhu, Yikun He

**Affiliations:** ^a^College of Life Sciences, Capital Normal University, Beijing 100048, PR China; ^b^Beijing Vocational College of Agriculture, Beijing 102442, PR China; ^c^Institute of Botany, Chinese Academy of Sciences, Beijing 100731, PR China

**Keywords:** nitric oxide, PIF3, PHYB, RGL3, root growth inhibition.

## Abstract

PHYTOCHROME INTERACTING FACTOR3 (PIF3) is an important component in the phytochrome signaling pathway and mediates plant responses to various environmental conditions. We found that PIF3 is involved in the inhibition of root growth of *Arabidopsis thaliana* seedlings induced by nitric oxide (NO) in light. Overexpression of *PIF3* partially alleviated the inhibitory effect of NO on root growth, whereas the *pif3-1* mutant displayed enhanced sensitivity to NO in terms of root growth. During phytochrome signaling, the photoreceptor PHYB mediates the degradation of PIF3. We found that the *phyB-9* mutant had a similar phenotype to that of *PIF3ox* in terms of responsiveness to NO. Furthermore, NO treatment promoted the accumulation of PHYB, and thus reduced PIF3 content. Our results further show that the activity of PIF3 is regulated by the DELLA protein RGL3[*RGA* (repressor of *ga1-3*) *LIKE 3*]. Therefore, we speculate that *PIF3* lies downstream of *PHYB* and *RGL3*, and plays an important role in the inhibitory effect of NO on root growth of *Arabidopsis* seedlings in light.

## SUMMARY

Nitric oxide (NO) promotes the accumulation of PHYB and thus reduces PIF3. The activity of PIF3 was also regulated by RGL3. *PIF3* may lie downstream of *PHYB* and *RGL3*, and regulate NO-induced root growth inhibition of *Arabidopsis* seedlings in light.

## INTRODUCTION

The growth and development of plants are influenced by various environmental factors, among which light is one of the most important, especially at the seedling stage. In the dark, seedlings undergo skotomorphogenesis, which leads to elongation of the hypocotyl and the presence of an apical hook and closed cotyledons (Alabadí and Blázquez, 2009). By contrast, photomorphogenesis in the light is characterized by reduced hypocotyl growth, open cotyledons, and chlorophyll biosynthesis. Thus, in addition to providing energy, light acts as a signal regulating plant growth and development.

Phytochromes are plant receptors that perceive light signals. There are five types of phytochromes (PhyA, B, C, D, and E) in *Arabidopsis thaliana*. Among them, PhyA is considered to be light-labile, whereas PhyB–E are light-stable (Sharrock and Quail, 1989; Clark et al., 1994). Together, they regulate a variety of developmental processes, including seed germination, seedling growth, shade avoidance, and flowering (Franklin and Quail, 2010). In addition, recent work has shown that phytochromes are involved in the light-induced promotion of root elongation by the ARP2/3–SCAR complex, a highly conserved nucleator of F-actin (Dyachok et al., 2011), and that root development is subject to regulation by a phytochrome-interacting ROP guanine–nucleotide exchange factor (Shin et al., 2010). Under light, phytochrome located in cytosol is activated and transferred into nucleus. There, it interacts with PIF proteins such as PIF3, causing their phosphorylation and subsequent degradation by the ubiquitin/26S proteasome. The activities of other types of PIF proteins, such as PIF1, PIF4, and PIF5, are regulated by phytochrome in a similar manner (Liu et al., 2011).

The first PIF family protein identified, PIF3, can interact with light-activated PHYA and PHYB (Ni et al., 1998; Stephenson et al., 2009). Large-scale genome sequencing has revealed several other homologous PIF proteins, including PIF1, PIF4, PIF5, and PIF6. These proteins can interact with different types of phytochrome to regulate photomorphogenesis and plant growth and development (Yamashino et al., 2003; Huq et al., 2004; Khanna et al., 2004). PIF family proteins are highly conserved in terms of sequence and domain structure, but, although *pif* single mutants share similar morphological characteristics, they also display unique phenotypes. This indicates that the functions of *PIF* genes are not completely redundant, and that each likely has its own unique biological roles (Leivar and Quail, 2011). For example, PIF1, PIF3, PIF4, and PIF5 all play parts in seedling skotomorphogenesis, but they also separately function in seed germination, chloroplast development, response to high temperature, and shade avoidance (Oh et al., 2004; Lorrain et al., 2008; Koini et al., 2009; Stephenson et al., 2009).

According to recent studies, PIF3 and PIF4 are regulated by gibberellin (GA) signaling (Lau and Deng, 2010), which also plays an important role in plant growth and development. DELLA protein family members are repressors involved in GA signaling and include five *Arabidopsis* proteins, namely GAI, RGA, RGL1, RGL2, and RGL3. DELLAs mediate many physiological effects of GA signaling, including in seed germination, elongation of stem and root, flowering, fruit, and seed development (Olszewski et al., 2002). DELLAs inhibit hypocotyl growth by inhibiting transcription activity of PIF3 and PIF4 (de Lucas et al., 2008; Feng et al., 2008). In addition, recent reports point to a role for nitric oxide (NO) in regulation of cell elongation through DELLA repressors during hypocotyl elongation and primary root growth (Lozano-Juste and León, 2011; Fernández-Marcos et al., 2012).

As an important signaling molecule, NO affects processes such as seed germination, photomorphogenesis, hypocotyl and root growth and development, flowering, senescence, and stress responses (Wendehenne et al., 2004; Crawford and Guo, 2005; Lamotte et al., 2005; Lozano-Juste and León, 2011). NO has a dose-dependent effect on root growth, wherein low concentrations of NO can promote the growth of plant roots, while high concentrations of NO suppress root growth (Gouvêa et al., 1997; Pagnussat et al., 2002; He et al., 2004). NO is likely to function downstream of auxin to regulate lateral root development (Pagnussat et al., 2003; Guo et al., 2008; Lanteri et al., 2008), and primary root growth inhibition exerted by NO may be at least partially explained by a reduction of auxin transport and response through a PIN1-mediated mechanism (Fernández-Marcos et al., 2011).

In contrast to our understanding of NO function in promoting lateral root emergence and development (Guo et al., 2008), the underlying mechanism explaining how primary root growth is controlled by NO along with phytochromes and hormones remains unknown. Here, we report that PIF3, through promoting both PHYB and RGL3 accumulation, partly mediates the inhibition of primary root growth by NO.

## RESULTS

### NO Inhibits Root Growth of *Arabidopsis* Seedlings in the Light

**Figure 1.**
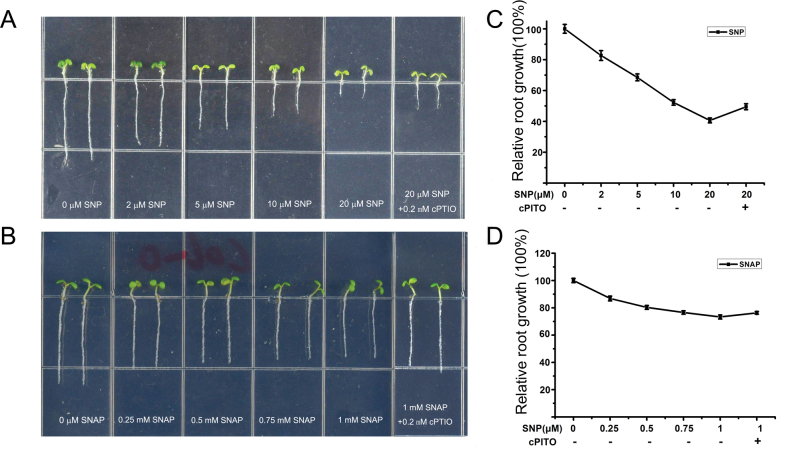
Response of Wild-Type (Col-0) to NO in Continuous Light. **(A)** Wild-type (WT) on growth medium supplemented with 0, 2, 5, 10, or 20 μM SNP or 20 μM SNP plus 0.2mM NO scavenger cPTIO. **(B)** WT on growth medium supplemented with 0, 0.25, 0.5, 0.75, or 1mM SNAP, or 1mM SNAP plus 0.2mM NO scavenger cPTIO. **(C)** Root length measurement of WT seedlings treated as described in (A). **(D)** Root length measurement of WT seedlings treated as described in (B). In (C) and (D), root length of control seedlings (no SNP) was set to 100%. Mean values and S.E. were calculated from at least 25 seedlings.

To test the effect of NO on root growth, we used two different NO donors. Sodium nitroprusside (SNP) was added to the upper cover inside of plates on which seedlings were grown in order to avoid possible side effects of the donor chemical (Supplemental Figure 1), whereas S-nitroso-N-acetylpenicillamine (SNAP) was added to the growth medium. Both SNP and SNAP inhibited root growth in a dose-dependent manner, and the inhibition could be rescued by the addition of the NO-specific scavenger 2-(4-carboxyphenylalanine) 4,4,5,5tetramethylimidazoline-1-oxyl-3-oxide (cPTIO). However, the effect of SNP on plant growth was stronger than that of SNAP. Based on these observations, we used SNP for further experiments (Figure 1).

### PIF3 Is Involved in the Effect of NO on Root Growth

**Figure 2.**
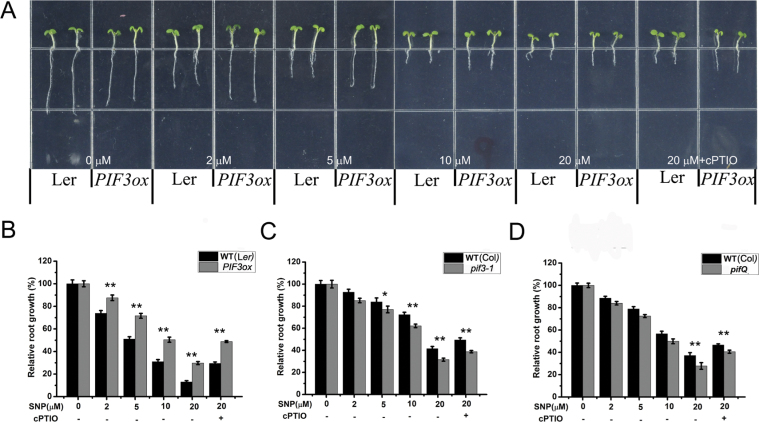
Responses of *PIF3ox*, *pif3-1*, and *pifQ* Mutants to NO under Continuous Light. **(A)** Wild-type (WT) (Ler) and *PIF3ox* seedlings growing on medium supplemented with SNP or 20 μM SNP plus 0.2mM cPTIO as indicated. **(B)** Root length of WT and *PIF3ox* seedlings treated as in (A). **(C)** Root length of WT (Col-0) and *pif3-1* seedlings treated with NO as in (A). **(D)** Root length of WT (Col-0) and *pifQ* seedlings treated with NO as in (A). In (B), (C), and (D), root length of control seedlings (no SNP) was set to 100%. Mean values and S.E. were calculated from at least 25 seedlings. Significant differences (*t*-test) compared to wild-type under the same conditions are indicated by asterisks: * *P* < 0.01; ** *P* < 0.001. *PIF3ox*, *35S::PIF3-HIS-MYC*. *pifQ*, *pif1-1/pif3-7/pif4-2/pil6-1*.

In order to study the role of NO in the root growth of *Arabidopsis* seedlings, we planted vernalized seeds of wild-type (WT), a *PIF3* overexpression line (*PIF3ox*), and *pif* mutant lines on growth medium supplied with 0, 2, 5, 10, or 20 μM SNP or 20 μM SNP with cPTIO. After 5 d, the root length of the seedlings was measured. NO markedly inhibited root growth of the wild-type in a dose-dependent manner (Figure 2A). However, the *PIF3ox* line was partially insensitive to NO, especially at high NO concentrations (Figure 2A, 2B, and Supplemental Figure 2A). The *pif3-1* mutant showed hypersensitivity to NO (Figure 2C), and the responses of the *pifQ* mutant (defective in PIF1, PIF3, PIF4, and PIF5) to NO were similar to those of *pif3-1* (Figure 2D). These results indicate that, as an important transcription factor regulating cell growth, PIF3 is involved in the inhibition by NO of root growth of *Arabidopsis* seedlings.

We also examined the individual roles of *PIF1*, *PIF4*, and *PIF5* in the NO-induced inhibition. Although these genes belong to the same transcription factor family, the root growth in overexpression lines of *PIF1*, *PIF4*, and *PIF5*, namely *PIF1ox*, *PIF4ox*, and *PIF5ox* seedlings, were basically the same as that of the WT (Supplemental Figure 3A–3C). Similarly, the loss-of-function mutants of PIF1, PIF4, and PIF5, namely *pif1-1*, *pif4-2*, and *pil6-1*, also showed no difference from WT (Supplemental Figure 3D–3F). Therefore, PIF1, PIF4, and PIF5 appear not to be involved in NO inhibition of root growth of *Arabidopsis* seedlings.

### Effect of Exogenous NO on PIF3 Expression

**Figure 3.**
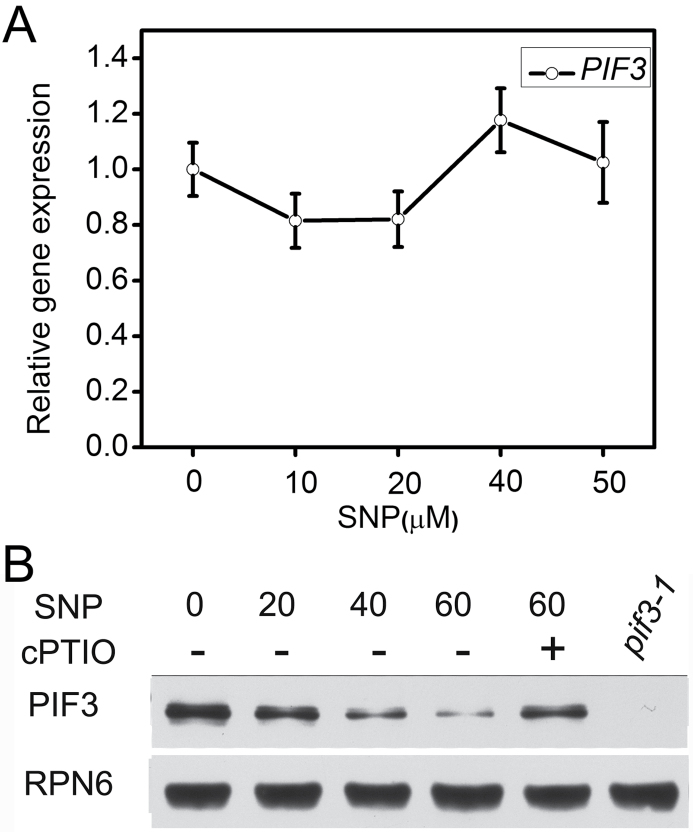
Effect of NO on *PIF3* Expression. **(A)** Quantitative RT–PCR was used to measure *PIF3* transcript levels. Total RNA was isolated from roots of wild-type (WT) seedlings treated with the indicated amounts of SNP for 6 h. Expression values for *PIF3* were calculated using the equation 2-ΔΔCt, and *18S* rRNA served as the control (Livak and Schmittgen, 2001; Pfaffl, 2001). Mean values and S.E. were calculated from at least three independent replicates. **(B)** Immunodetection of PIF3 in roots of WT seedlings treated with SNP as in (A). RPN6 was used as a loading control.

NO could regulate the root growth of *Arabidopsis* seedlings through PIF3 by reducing PIF3 transcript and/or protein accumulation. We therefore examined the effects of NO on *PIF3* transcript and protein levels. Real-time PCR results indicated that NO had little effect on *PIF3* transcript accumulation (Figure 3A). However, treatment with exogenous NO significantly reduced the amount of PIF3 in roots (Figure 3B). Therefore, the effect of NO on PIF3 appears to be mainly at the protein level.

### NO Promotes the Accumulation of PHYB Upstream of PIF3

**Figure 4.**
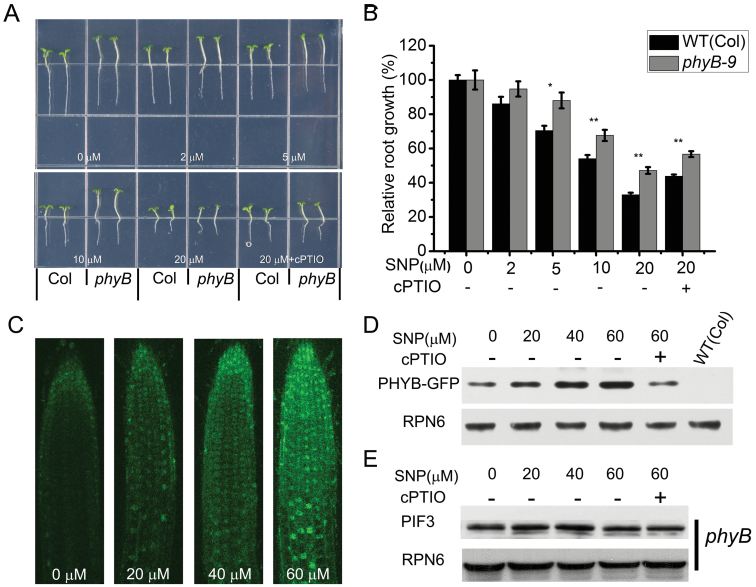
PHYB Mediates NO-Induced Root Growth Inhibition. **(A)** Wild-type (WT) and *phyB-9* mutant growth on medium supplemented with SNP or 20 μM SNP plus 0.2mM cPTIO as indicated. **(B)** Measurement of root length. Root length without NO treatment was set to 100%. Mean values and S.E. were calculated from at least 25 seedlings. Significant differences (*t*-test) compared with wild-type under the same conditions are indicated by asterisks: * *P* < 0.01; ** *P* < 0.001. **(C)** GFP fluorescence in root tips of 5-day-old *35S::PHYB–GFP* seedlings after 6-h treatment with the indicated amounts of SNP. **(D)** Immunoblot showing PHYB–GFP in *35S::PHYB–GFP* seedlings treated as in (A). **(E)** Immunodetection of PIF3 in roots of *phyB* mutant seedlings treated with SNP as in (A). RPN6 served as loading control.

PHYB directly interacts with PIF3 to mediate PIF3 degradation and initiate several photomorphogenic processes (Park et al., 2004). In order to detect whether PIF3’s effect on root growth upon NO treatment is related to PHYB, we investigated root growth in the loss-of-function *phyB-9* mutant treated with different amounts of NO. The root growth in *phyB-9* was less sensitive to NO compared to that of WT (Figure 4A, 4B, and Supplemental Figure 2B), suggesting that the effect of *PIF3* on root growth involves PHYB.

If the root growth inhibition by PIF3 is associated with PHYB action, then the PIF3 level should be directly related to the protein content of PHYB. In order to detect effects of NO on PHYB content, we monitored PHYB–GFP in root tip cells of 5-day-old seedlings after treatment with SNP for 6h. PHYB–GFP signal became stronger with increased SNP concentration (Figure 4C). Immunoblot analysis also showed that NO promoted the accumulation of PHYB–GFP (Figure 4D). In contrast to the reduction in PIF3 observed in WT seedling roots treated with exogenous NO (Figure 3B), PIF3 accumulation did not change in *phyB-9* mutant seedlings treated with exogenous NO, indicating that the effects of NO on PIF3 are mediated by PHYB.

### The Effect of PIF3 on Root Growth Is Affected by RGL3

**Figure 5.**
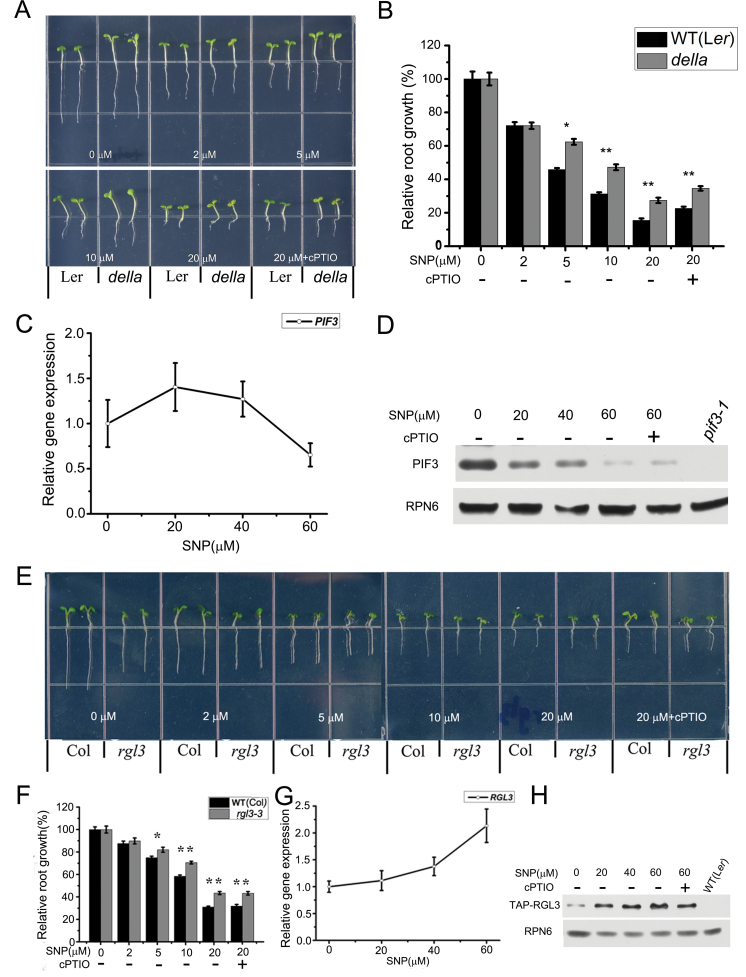
RGL3 Is Involved in NO-Induced Root Growth Inhibition. **(A)** Wild-type (WT) and *della* growth on medium supplemented with SNP or 20 μM SNP plus 0.2mM cPTIO as indicated. **(B)** Measurement of root length. Root length of untreated seedlings was set to 100% for each genotype. *della*, *gai-t6/rga-t2/rgl1-1/rgl2-1/rgl3-1*. Mean values and S.E. were calculated from at least 25 seedlings. Significant differences (*t*-test) compared with wild-type under the same conditions are indicated by asterisks: * *P* < 0.01; ** *P* < 0.001. **(C)** Quantitative RT–PCR was used to measure *PIF3* transcript levels. Total RNA was isolated from roots of *della* mutant seedlings treated with the indicated amounts of SNP for 6 h. Expression values of PIF3 were calculated using the equation 2-ΔΔCt, and *18S* rRNA served as the control. Mean values and S.E. were calculated from at least three independent replicates. **(D)** Immunodetection of PIF3 in roots of *della* mutant seedlings treated with SNP as in (C). RPN6 was used as a loading control. **(E)** WT and *rgl3* growth on medium supplemented with SNP or 20 μM SNP plus 0.2mM cPTIO as indicated. **(F)** Measurement of root length. Root length of untreated seedlings was set to 100% for each genotype. Mean values and S.E. were calculated from at least 25 seedlings. Significant differences (*t*-test) compared with wild-type under the same conditions are indicated by asterisks: * *P* < 0.01; ** *P* < 0.001. **(G)** Measurement of *RGL3* expression by quantitative RT–PCR. Seedlings were treated with SNP at the indicated concentrations for 6 h. *18s rRNA* transcripts provided loading control. Mean values and S.E. were calculated from at least three independent replicates. **(H)** Immunodetection of RGL3 in roots of 35S::TAP-RGL3 treated with SNP for 6 h. RPN6 served as loading control.

Recent studies showed that DELLAs interact with PIFs and inhibit the regulatory function of PIF transcription factors on downstream gene expression (Olszewski et al., 2002; Lau and Deng, 2010). To detect whether the inhibitory effect of PIF3 on root growth is associated with DELLA activity, we examined root growth of a mutant lacking five DELLAs (*della*, *gai-t6/rga-t2/rgl1-1/rgl2-1/rgl3-1*, Figure 5A–5D). The sensitivity of the *della* mutant to NO was decreased compared to that of the wild-type (Figure 5A and 5B). However, both the transcript and protein levels of *PIF3* in *della* mutant showed little change under exogenous NO treatment (Figure 5C and 5D), indicating that the activity, but not the amount, of *PIF3* was affected by RGL3.

Root growth of a mutant lacking four DELLA proteins (*tetra*, *gai-t6/rga-t2/rgl1-1/rgl2-1*) in the presence of different concentrations of NO was almost the same as that of the WT (Supplemental Figure 4A and 4B). The difference between *tetra* and *della* is the presence of *RGL3*, so we examined the role of RGL3 in NO responses (Figure 5E–5H). Root growth of the *rgl3-1* mutant was less sensitive to NO (Figure 5E, 5F, and Supplemental Figure 2C), and NO significantly promoted RGL3 accumulation at both the transcription and protein levels (Figure 5G and 5H). These results suggest that the function of PIF3 in inhibiting root growth is also regulated by RGL3 in response to NO.

### Inhibitory Effect of NO on Root Growth of *Arabidopsis* Seedlings Is Less Affected by PIF3 in the Dark

**Figure 6.**
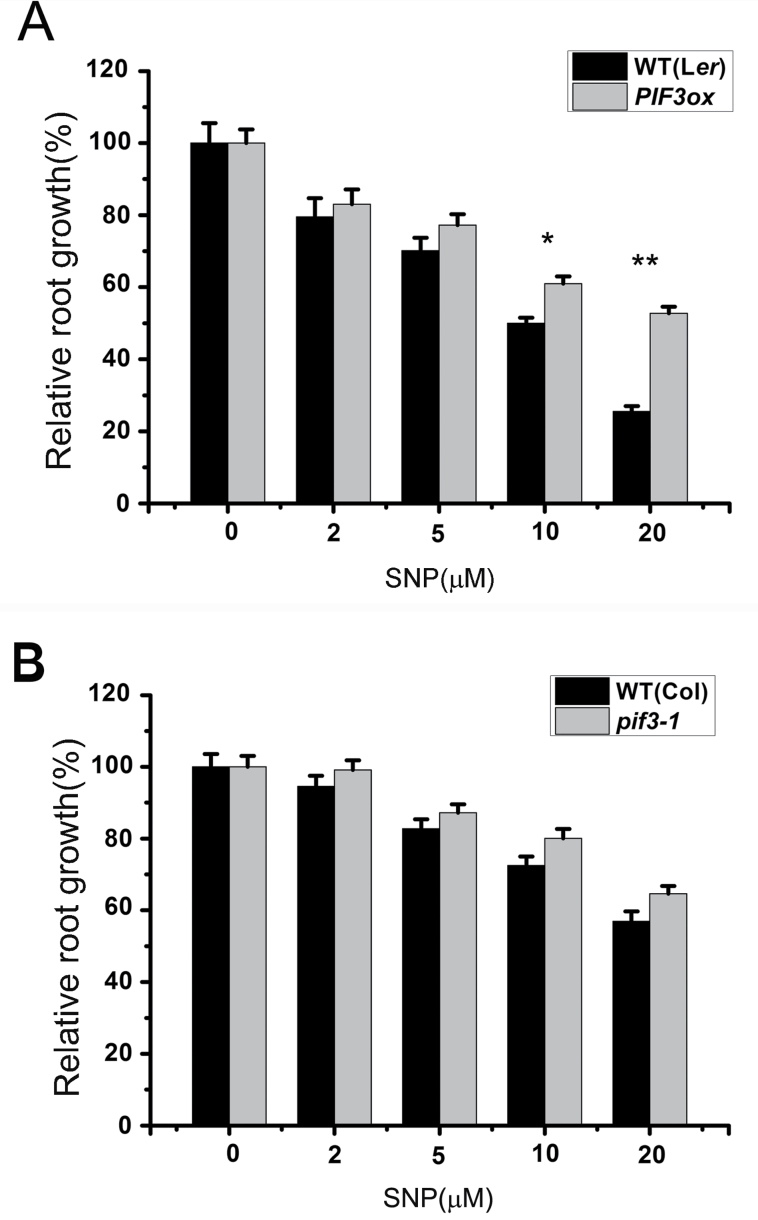
Response of *PIF3ox* and *pif3-1* to NO in the Dark. **(A)** Measurement of root length in wild-type (WT) and *PIF3ox* treated with the indicated amounts of SNP. **(B)** Measurement of root length in WT and *pif3-1* treated with SNP. Root length of untreated seedlings was set to 100% for each genotype. *PIF3ox*, *35S::PIF3-HIS-MYC*. Mean values and S.E. were calculated from at least 25 seedlings. Significant differences (*t*-test) compared to wild-type under the same conditions are indicated by asterisks: * *P* < 0.01; ** *P* < 0.001.

We then examined the effect of NO on root growth of *Arabidopsis* seedlings in the dark. Seeds were placed on the growth medium with SNP on the upper cover inside of the plates and, after exposure to light for 24h to promote the full release of NO, they were incubated in the dark for 5 d. NO still inhibited root growth under these conditions. Although overexpression of *PIF3* could partially restore the effect of NO on root growth in the dark (Figure 6A, Lersburg ecotype), the *pif3-1* mutant did not show the NO-sensitive phenotype (Figure 6B, Columbia ecotype). The impact of PIF3 on NO inhibition of root growth in the dark needs to be further investigated.

## DISCUSSION

Previous studies have shown that PIF transcription factors, as core components of phytochrome signaling, mediate plant growth and developmental processes such as seed germination, seedling photomorphogenesis, shade avoidance, and flowering (Park et al., 2004; Castillon et al., 2007). The important signaling molecule NO also affects plant growth and development (Gouvêa et al., 1997; Pagnussat et al., 2002; He et al., 2004). Here, we found that overexpression of *PIF3* could counteract the inhibitory effect of NO on root growth in seedlings (Figure 2A and 2B), whereas the root growth of the *pif3* mutant was reduced (Figure 2C). There are different responses to SNP at lower concentration between *pif3* and *pifQ*, because *pifQ* mutant (defective in *PIF1*, *PIF3*, *PIF4*, and *PIF5*) shows combination phenotypes of *PIF1*, *PIF4*, and *PIF5* that may play different roles in regulation of root growth (Figure 2D). These results indicate that NO inhibition of root growth might be achieved through a reduction in *PIF3* expression. Consistently with this, we found that the level of PIF3 in roots was significantly reduced upon treatment with exogenous NO (Figure 3B). The comparatively small changes in *PIF3* transcript accumulation (Figure 3A) indicate that the effect of NO on PIF3 occurs primarily at the posttranscriptional level. Our data suggest that PIF3 plays an important role in integrating NO and light signals.

Phytochromes have been reported to be involved in regulation of root growth. For instance, phytochromes contribute to light-induced promotion of root elongation by suppressing COP1-mediated SCAR1 degradation (Dyachok et al., 2011). In addition, the phytochrome-interacting protein PIRF1 has been reported to be a negative regulator of phytochrome-mediated root growth and development in *Arabidopsis* (Shin et al., 2010). PIF3 is a key component in phytochrome signaling and its activity is directly regulated by PHYB, which promotes the degradation of PIF3, thus inhibiting its activity (Leivar and Quail, 2011). During *Arabidopsis* seedling growth and development, overexpression of *PIF3* inhibits root growth, leading to a phenotype similar to that of the *phyB* mutant (Correll and Kiss, 2005). Our results show that PIF3 protein levels did not change in the *phyB* mutant in response to NO (Figure 4E), consistently with the previous report. On the other hand, *PIF3ox* and *phyB* showed a similar decreased sensitivity to NO in terms of root growth (Figures 2A and 4A). We also found that NO promotes the accumulation of PHYB (Figure 4C and 4D). Therefore, we speculate that PHYB–PIF3 might be involved in the process of NO inhibition of root growth in *Arabidopsis*.

Plant hormones also play an important role in NO-induced root growth inhibition. Gibberellin signaling regulates NO-induced root growth inhibition in the differentiation zone, mainly though DELLA repressors (Fernández-Marcos et al., 2012). A recent study demonstrated that NO induces photomorphogenesis by promoting DELLA accumulation and decreasing *PIF* expression, especially in the inhibition of hypocotyl elongation (Lozano-Juste and León, 2011). Since the activity of PIF3 may be regulated by DELLAs, we examined the role of DELLA proteins in the inhibitory effect of NO on root growth of *Arabidopsis* seedlings. Genetic analysis suggested that only one DELLA protein, RGL3, was involved in the response of root growth to NO. Consistently with this, RGL3 accumulated in the roots in response to NO treatment (Figure 5H). Thus, PIF3 is a likely targeted by RGL3 in NO-induced root growth inhibition.

**Figure 7.**
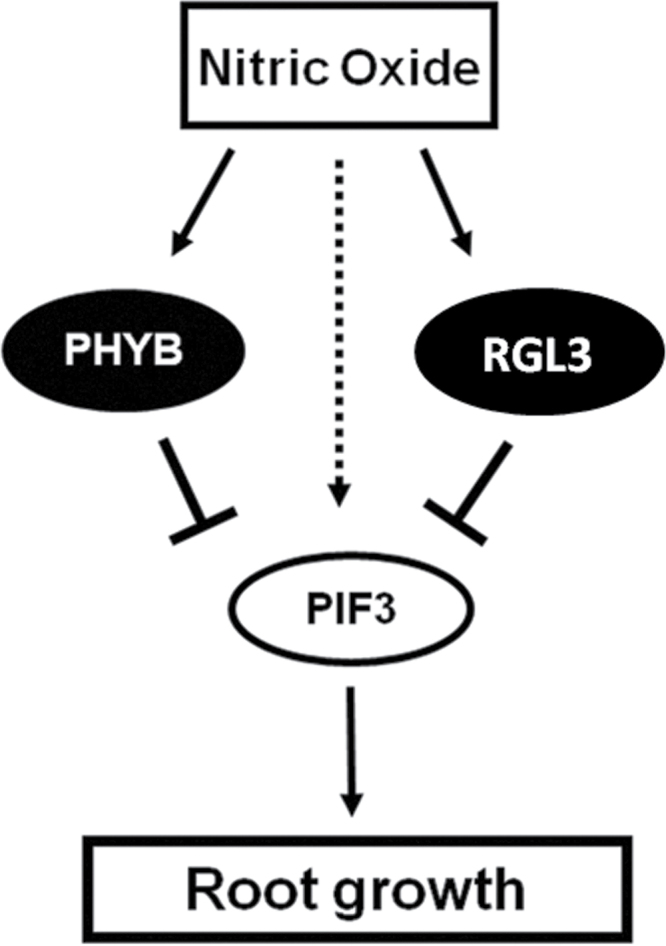
A Scheme of PIF3 in Regulation of Primary Root Growth under Nitric Oxide Conditions. The proposed mechanism points the central role of PIF3 in the regulation of root growth in a process that is mediated by both PHYB and RGL3.

Together, this work points the central role of PIF3 in the regulation of root growth by NO in a process that is mediated by both PHYB and RGL3 (Figure 7). This study sheds light on crosstalk among NO, light, and gibberellin in control of root growth.

## METHODS

### Plant Materials and Growth Conditions

The WT *Arabidopsis thaliana* ecotypes used in this study were Columbia-0 (Col-0) and Landsberg *erecta* (L*er*). The *35S::PIF1-MYC*, *35S::PIF3-HIS-MYC*, *35S::PIF4*, *35S::PIF5*, *pif1-1*, *pif3-1*, *pif4-2*, *pil6-1*, *pifQ* (*pif1-1 pif3-7 pif4-2 pil6-1*), *phyB-9*, *tetra* (*gai-t6 rga-t2 rgl1-1 rgl2-1*), *della* (*gai-t6 rga-t2 rgl1-1 rgl2-1 rgl3-1*), *35S::TAP-RGL3*, *rgl3*, and *35S::phyB-GFP*, *phyB-9* lines were described previously (Dill et al., 2001; Bauer et al., 2004; Achard et al., 2007; Feng et al., 2008; Niwa et al., 2009; Leivar and Quail, 2011). All seeds were surface-sterilized with 10% (v/v) bleach solution for about 10 min, rinsed with sterile water at least four times, and then sown on half-strength Murashige and Skoog (1/2 MS) medium containing 0.8% (w/v) agar. After 4 d at 4°C in the dark to synchronize germination, the plates were transferred to a growth chamber with continuous white light (about 100 μmol m^–2^ s^–1^) and maintained at 23°C for 5 d.

### NO Treatment

To avoid possible effects of chemicals other than NO gas on root growth, NO treatments were performed by photochemically mediated release of NO gas from the NO donor sodium nitroprusside (SNP), which was mixed with a small amount of growth medium before solidification and added inside the upper cover of the plates at the desired concentrations (Supplemental Figure 1; Bai et al., 2012). Seedlings grew on SNP-free agar medium in the lower portion of the container. In this way, we could ensure that the effect on the seedlings was due to NO rather than to SNP. Another NO donor, S-nitroso-N-acetylpenicillamine (SNAP), which has few side effects (Pereira et al., 2011), was used to confirm the effects of NO on plant growth. The NO-specific scavenger 2-(4-carboxyphenylalanine) 4,4,5,5tetramethylimidazoline-1-oxyl-3-oxide (cPTIO) was added in the growth medium as indicated. For root growth assays, SNP treatment was applied from seed germination to the end point of the experiment. For gene expression and protein analysis, a 6-h treatment time was used.

### Root Length Measurement

After the indicated time of growth and treatment, at least 25 seedlings were laid horizontally on the agar plates, digital pictures were taken, and hypocotyl length was measured using a standard 5-mm-scaled ruler with ImageJ software (Abramoff et al., 2004).

### Transcript Analysis

Total RNA was isolated from roots of 5-day-old seedlings and further analyzed by quantitative real-time PCR as described previously (Jiang et al., 2007). The primer pairs used for PCR amplification as follows: *PIF3*, 5′-CCACGGACCACAGTTC CAAG-3′ and 5′-ATCGCCACTGGTTGTTGTTG-3′; *18S rRNA*, 5′-ATACGTGCAACAAACCC-3′ and 5′-CTACCTCCCCGTGTCA-3′.

### Microscopy

Confocal micrographs were obtained with a Leica TCS SP2 inverted confocal laser microscope (Leica). The excitation wavelength for GFP detection was 488 nm. All images were obtained with the same setting and intensity parameters. PHYB–GFP fluorescence was observed in the tip of the primary root of 5-day-old *35S::PHYB–GFP* seedlings.

### Immunoblot Analysis

Roots were harvested and ground into fine powder in liquid nitrogen. Total proteins were subsequently extracted in extraction buffer containing 25mM Tris–HCl, pH 7.5, 10mM NaCl, 10mM MgCl_2_, 4mM PMSF, and 5mM DTT as previously described (Wang et al., 2009). Cell debris was removed by two 10-min centrifugations of 17 000 *g* at 4°C. The supernatant was collected, and protein concentration was determined by the Bio-Rad protein assay. All harvested protein samples were resolved on 8% SDS–PAGE gels and transferred to polyvinylidene fluoride membranes. Anti-PIF3 polyclonal antibody at 1:200 (v/v) dilution (gift from Professor Deng, Yale University), anti-GFP monoclonal antibody at 1:5000 (v/v) dilution (Abmart), anti-Myc monoclonal antibody at 1:1000 (v/v) dilution (Sigma-Aldrich), and anti-RPN6 polyclonal antibody at 1:3000 dilution (Millipore) were used as primary antibodies. Immunoblots were detected using peroxidase-conjugated anti-mouse and anti-rabbit IgG (Sigma-Aldrich) at 1:8000 (v/v) dilutions and ECL Plus reagent (GE Healthcare).

## SUPPLEMENTARY DATA

### Supplementary Data are available at *Molecular Plant Online.*

## FUNDING

This work was supported by the National Natural Science Foundation of China (No. 30771094 and 31270821 to S.B.), the Beijing Natural Science Foundation (No. 5112006 to S.B.), and the Ministry of Science and Technology of China (No. 2013CB967300 to Y.H.).

## Supplementary Material

Supplementary Data
