# Supplementary material for: PIF3 Is Involved in the Primary Root Growth Inhibition of Arabidopsis Induced by Nitric Oxide in the Light
Source: Mol Plant. 2013 Oct 21;7(4):616–25. doi: 10.1093/mp/sst142 (PMC3973492; doi:10.1093/mp/sst142)
Supplement: Supplementary Data [file supp_7_4_616__index.html]

PIF3 Is Involved in the Primary Root Growth Inhibition of Arabidopsis Induced by Nitric Oxide in the Light — PIF3 Is Involved in the Primary Root Growth Inhibition of Arabidopsis Induced by Nitric Oxide in the Light — PIF3 Is Involved in the Primary Root Growth Inhibition of Arabidopsis Induced by Nitric Oxide in the Light — Supplementary Data 

# PIF3 Is Involved in the Primary Root Growth Inhibition of *Arabidopsis* Induced by Nitric Oxide in the Light

## Supplementary Data

Data files

**Files in this Data Supplement:**

- Supplementary Data - Supplementary Data
