# Supplementary material for: PIF3 Is Involved in the Primary Root Growth Inhibition of Arabidopsis Induced by Nitric Oxide in the Light
Source: Mol Plant. 2013 Oct 21;7(4):616–25. doi: 10.1093/mp/sst142 (PMC3973492; doi:10.1093/mp/sst142)
Supplement: Supplementary Data [file supp_sst142_supplemental_file.doc]

Supplemental Figures S1-S4


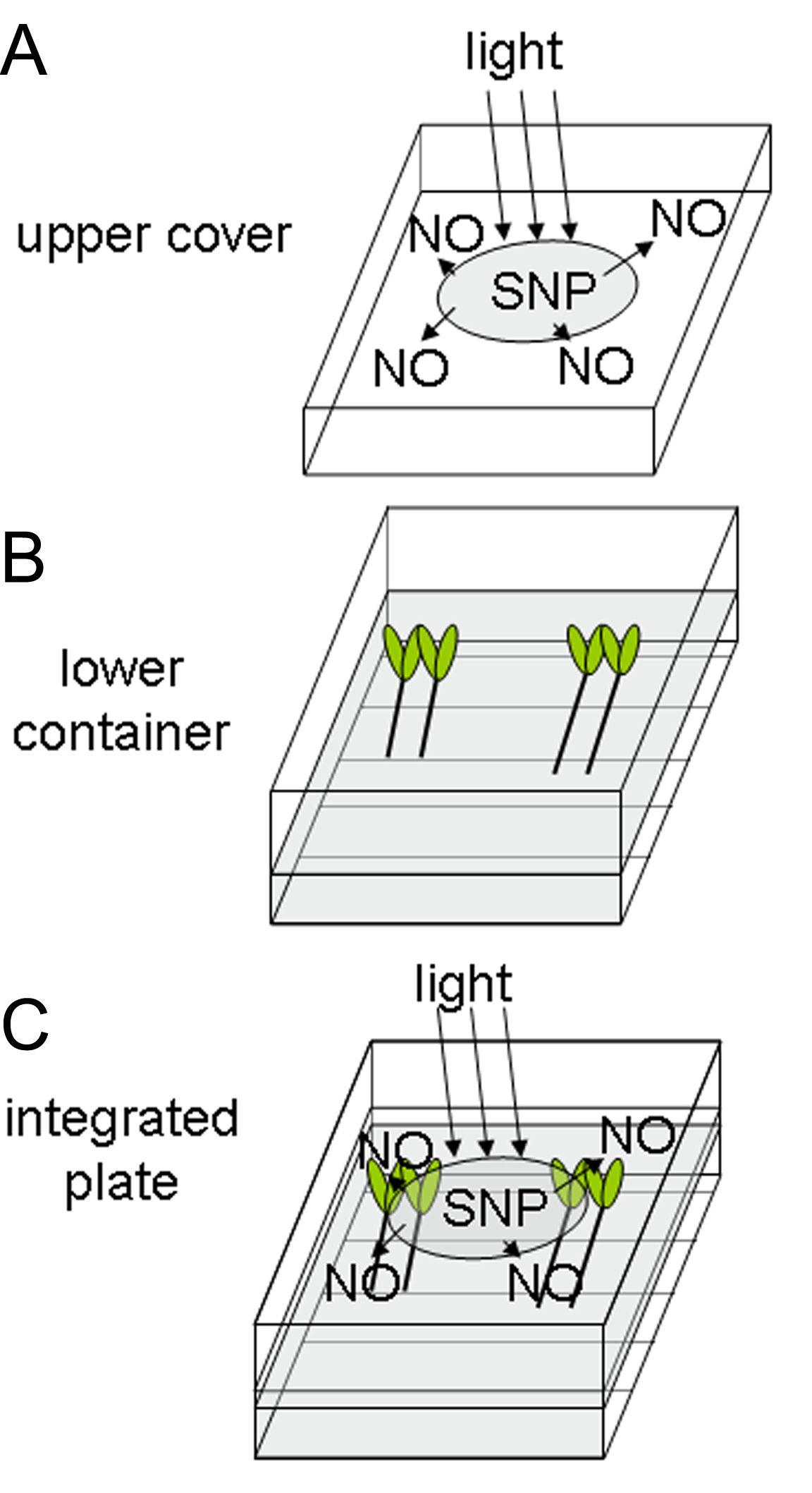


Figure S1. SNP treatment method. A, SNP was mixed with a small amount of growth medium before it solidified and added inside the upper cover of the plate to supply NO gas under light. B, Seedlings grew on growth medium without SNP in the lower portion of the container to avoid contacting the SNP chemical directly. C, Upon light activation, NO is released inside the plates. The effect to seedlings is from NO rather than SNP.


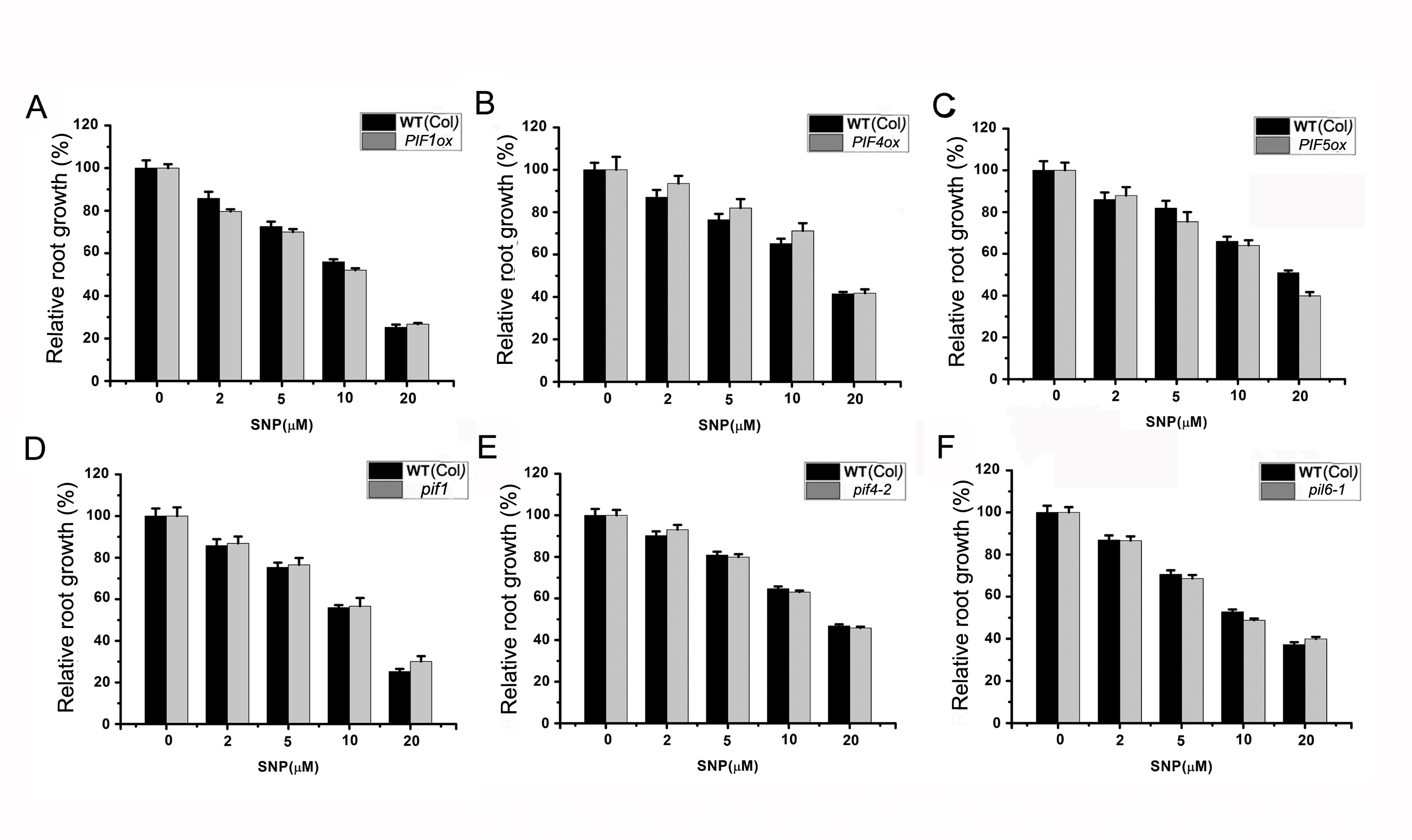


Figure S2. PIF1, PIF4 and PIF5 are not involved in NO-induced root growth inhibition. Root length of seedlings of WT and *PIF1ox* (A), *PIF4ox* (B), *PIF5ox* (C), *pif1* (D), *pif4-2* (E), *pil6-1* (F). A-F, Seedlings were treated with SNP as indicated. Root length of control seedlings (no SNP) was set to 100%. Mean values and S.E. were calculated from at least 25 seedlings. Significant differences (*t* test) compared with wild type under the same conditions are indicated by asterisks: *, P < 0.01; **, P < 0.001. *PIF1ox, 35S::PIF1-MYC; PIF4ox*, *35S::PIF4*; *PIF5ox*, *35S::PIF5*.


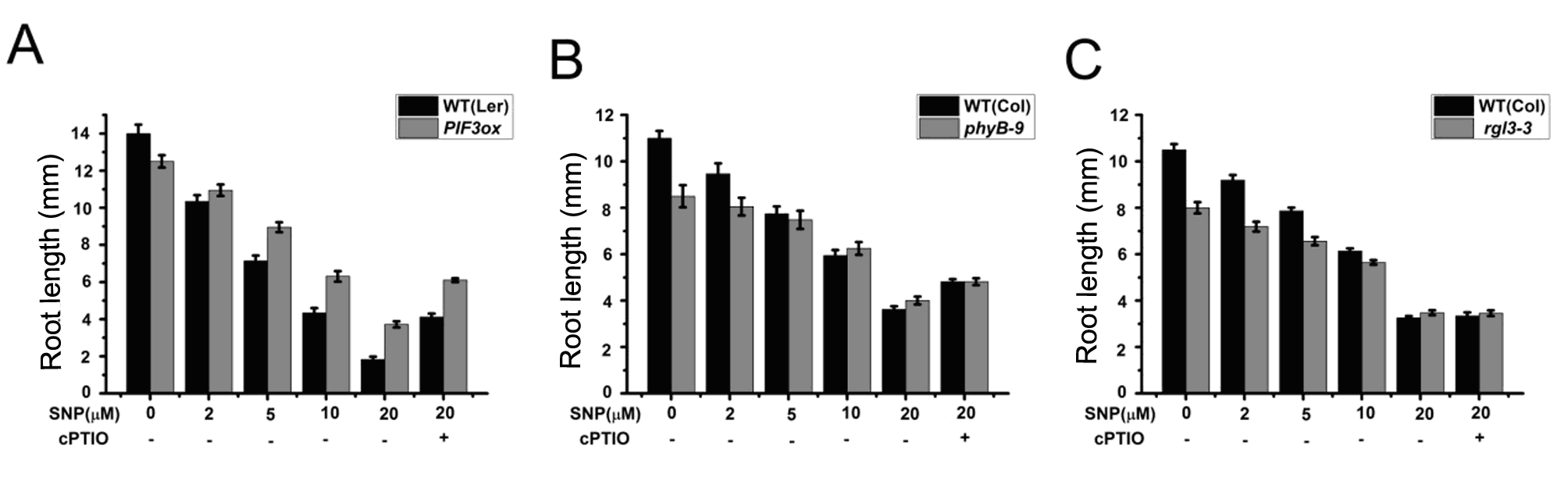


Figure S3. Responses of *PIF3ox*, *phyB-9* and *rgl3-3* mutants to NO under continuous light (absolute root lengths). A, Root length of WT (Ler) and PIF3ox seedlings growing on medium supplemented with 0, 2, 5, 10 and 20 μM SNP or 20 μM SNP plus 0.2 mM cPTIO as indicated. B, Root length of WT (Col-0) and *pif3-1* seedlings treated with NO as in A. C, Root length of WT (Col-0) and pifQ seedlings treated with NO as in A. Mean values and S.E. were calculated from at least 25 seedlings. *PIF3ox, 35S::PIF3-HIS-MYC*.


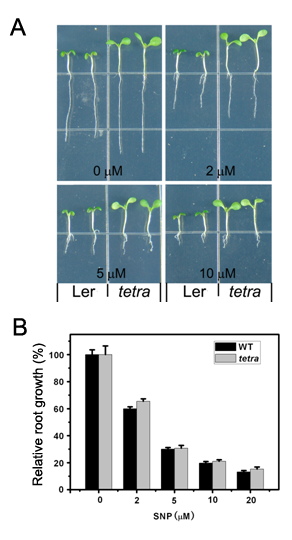


Figure S4. Four DELLA proteins are not involved in NO-induced root growth inhibition. A, WT and *tetra* seedlings on growth medium supplemented with the indicated amounts of SNP. B, Measurement of root length. Root length of untreated seedlings was set to 100% for each genotype. *tetra*, *gai-t6/rag-t2/rgl1-1/rgl2-1*. Mean values and S.E. were calculated from at least 25 seedlings. Significant differences (*t* test) compared with wild type under the same conditions are indicated by asterisks: *, P< 0.05; **, P < 0.01; ***, P < 0.001.
